# Role of Uromodulin in Salt-Sensitive Hypertension

**DOI:** 10.1161/HYPERTENSIONAHA.122.19888

**Published:** 2022-09-12

**Authors:** Sheon Mary, Philipp Boder, Sandosh Padmanabhan, Martin W. McBride, Delyth Graham, Christian Delles, Anna F. Dominiczak

**Affiliations:** School of Cardiovascular and Metabolic Health, University of Glasgow, Glasgow, United Kingdom.

**Keywords:** blood pressure, kidney, loop of Henle, sodium, uromodulin

## Abstract

The exclusive expression of uromodulin in the kidneys has made it an intriguing protein in kidney and cardiovascular research. Genome-wide association studies discovered variants of uromodulin that are associated with chronic kidney diseases and hypertension. Urinary and circulating uromodulin levels reflect kidney and cardiovascular health as well as overall mortality. More recently, Mendelian randomization studies have shown that genetically driven levels of uromodulin have a causal and adverse effect on kidney function. On a mechanistic level, salt sensitivity is an important factor in the pathophysiology of hypertension, and uromodulin is involved in salt reabsorption via the NKCC2 (Na^+^-K^+^-2Cl^−^ cotransporter) on epithelial cells of the ascending limb of loop of Henle. In this review, we provide an overview of the multifaceted physiology and pathophysiology of uromodulin including recent advances in its genetics; cellular trafficking; and mechanistic and clinical studies undertaken to understand the complex relationship between uromodulin, blood pressure, and kidney function. We focus on tubular sodium reabsorption as one of the best understood and pathophysiologically and clinically most important roles of uromodulin, which can lead to therapeutic interventions.

Uromodulin, or Tamm-Horsfall protein, is the most abundant protein secreted into the urine of healthy individuals and is primarily synthesized by the epithelial cells lining the kidney tubules.^1,2^ In 1987, Pennica et al^3^ demonstrated that Tamm-Horsfall protein is identical to uromodulin (UMOD). Around 90% of uromodulin is secreted by the epithelial cells lining the thick ascending limb (TAL) of Henle’s loop, with the remaining 10% by cells of the early distal convoluted tubule (DCT).^4^ Although uromodulin was first discovered in human urine, it has since been detected in the kidneys of all placental mammals.^5^ The human *UMOD* gene is highly conserved.^6^ Homologues are present in fish, amphibians, reptiles, birds, and mammals.^6^ There is also a high degree of cross-species conservation in amino acid sequence, including 95% similarity with 77% identity between rat and human and 79% similarity with 70% identity between mouse and human.^7^ This is important to consider when discussing uromodulin studies using animal models.

This review is designed to discuss the role of uromodulin in salt-sensitive hypertension utilizing the bench to bedside and back again approach. We aim to identify mechanisms that are clinically applicable and might improve treatment modalities for difficult-to-treat essential hypertension. We focus on possible genetic variants within the uromodulin gene, which could improve and widen treatment choices for hypertension management. For the purpose of this review, we use the term uromodulin for general discussions. In specific contexts, we use the abbreviations *UMOD* for the human uromodulin gene; *Umod* for the rodent uromodulin gene; and UMOD for uromodulin protein.

## Gene to Protein

**Figure 1.**
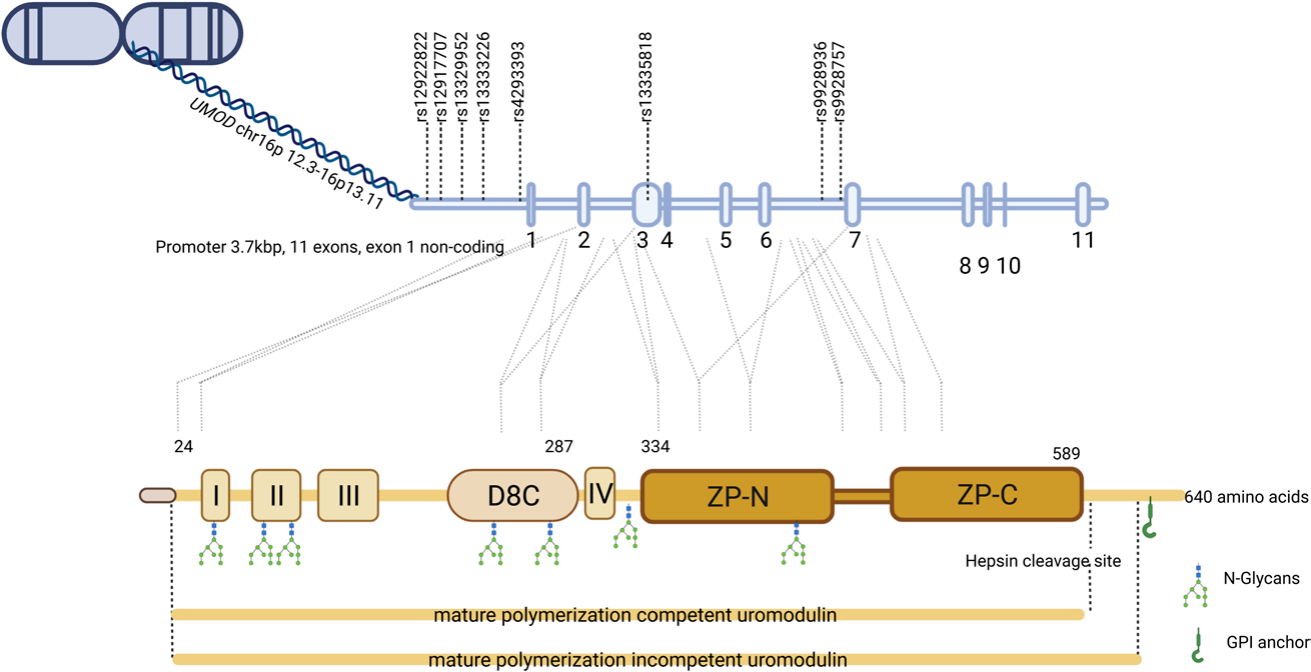
**Uromodulin gene to protein.** The 20 kb gene of *UMOD* is translated to 640 amino acid protein by 10 exons. Major single nucleotide polymorphisms are represented on the gene. The protein structure represented in image consists of an N-terminal signal peptide (1–24), 4 epidermal growth factor-like domains (I, II, III, and IV), a domain rich in 8 cysteine (D8C), the 2 zona pellucida domain (ZP-N and ZP-C), and a glycosylphosphatidylinositol (GPI) anchoring site.

The *UMOD* gene is located on chromosome 16p12.3 to 16p13.11 and comprises 11 exons, which extend over 20 kb (Figure 1).^8^ The regulation of *UMOD* expression is not well explored. Knockout studies in mice have confirmed that HNF1β (hepatocyte nuclear factor-1β) interacts with the *Umod* promoter at 2 distinct target sites and positively regulates its expression.^9^ Further in silico phylogenetic footprinting analyses and protein-protein interactions studies indicate a large network of transcription factors that could regulate uromodulin expression, however, further validation is needed.^10^

The uromodulin protein consists of 640 amino acids with 4 EGF (epidermal growth factor)-like domains, a domain rich in 8 cysteine (D8C), one bipartite zona pellucida-like domain (ZP-N and ZP-C), and a C-terminal patch of hydrophobic residues. Post-translational modifications of uromodulin include 8 N-glycosylation sites, 24 conserved disulfide bridges, and a C-terminal glycosylphosphatidylinositol anchor (Figure 1).^3,11^ It undergoes a series of modifications and cleavage during its maturation along the secretory pathway (Figure 1). This begins with the insertion into the endoplasmic reticulum (ER) using an N-terminal signal peptide sequence, which is cleaved during maturation.^12^ This is followed by the formation of the 24 intramolecular disulfide bonds in the oxidative environment of the ER.^13^ Correct tertiary folding is required for the release of UMOD from the ER, before a series of extensive glycan modifications in the Golgi. Glycosylation accounts for 30% of the molecular mass of UMOD and plays a key role in its function. High-mannose residues are converted to glycan which can be sialylated, fucosylated, or sulfated.^14^ There is also evidence for O-glycosylation of UMOD.^15^ The C-terminal glycosylphosphatidylinositol anchoring domain directs uromodulin to the membrane after cotranslational insertion into the ER. Uromodulin is secreted at 50 to 150 mg per 24 hours in humans and polymerizes to form a porous, 3-dimensional matrix-like structure.^16–21^ Protein polymerization involves the cleavage of membrane-bound uromodulin by the serine protease hepsin at a conserved cleavage site located at the C-terminal of a bipartite ZP domain.^22^ The process releases a polymerization inhibitory motif known as the external hydrophobic patch, which interacts with the internal hydrophobic patch.^23^ More specifically, mechanistic studies have shown that release of the external hydrophobic patch exposes a large hydrophobic interface on the internal hydrophobic patch for N-terminal ZP domain homodimerization.^24^ Cryo-electron tomography of the 3-dimensional structure of native uromodulin polymers demonstrated a zigzag-shaped backbone formed by polymerized ZP domains, forming modules of 8.5 nm in length, as well as protruding arms composed of the EGF and D8C domains.^25,26^ The external hydrophobic patch release following cleavage induces a major conformational change in the ZP-N/ZP-C linker region, linking 3 consecutive monomers. Most of the uromodulin is secreted into the tubular lumen via the apical membrane. However, a monomeric form is also secreted into the blood (circulating or serum uromodulin) via the basolateral membrane and is detected at levels 100- to 1000-fold lower than in urine.^27^ N-glycans are likely to play a key role in the polarized sorting of uromodulin^28,29^; however, further research into the precise mechanisms is warranted. Similarly, the trafficking proteins involved in the transport of uromodulin are also not well understood. NM2 (nonmuscle myosin II) motor proteins have been associated with vesicle biogenesis at the Golgi with different isoforms showing unique localization in the tubules.^30^ Conditional knockout experiments of NM2 isoforms *Myh9* and *Myh10* in the TAL of mice resulted in intracellular accumulation of uromodulin, suggesting a critical role in its localization.^31^

## Function in Ion Transport

The physiological functions of uromodulin are multifaceted and not yet fully understood. Uromodulin has well-defined roles in water homeostasis and urine concentration. Early studies demonstrated that uromodulin forms a hydrophobic, gel-like seal which may contribute to the water impermeability and the maintenance of counter-current gradients of the TAL.^32^ Experiments with conventional knockout mice (*Umod^−/−^*) showed impaired urinary concentration following water deprivation,^33^ highlighting its critical role in water reabsorption. The TAL region is involved in the reuptake of 30% of the filtered sodium, primarily via the apical NKCC2 (Na^+^-K^+^-2Cl^−^ cotransporter).^34^ The levels of surface NKCC2 decreased at the TAL apical membrane of conventional *Umod^−/−^* knockout mice.^33^ Instead, NKCC2 accumulated inside subapical vesicles, suggesting uromodulin is critical to its intracellular trafficking (detailed below).^35^ Uromodulin is also critically involved in the regulation of other ions and ion channels. The apically located renal outer medullary potassium channel (ROMK) controls K^+^ homeostasis in the TAL.^36^ It helps maintain the K^+^ conductance required for the functioning of NKCC2 and the lumen-positive voltage for paracellular reabsorption of other ions (eg, Ca^2+^ or Mg^2+^) by recycling K^+^ back into the tubular lumen. Interaction assays identified a potential association between ROMK and uromodulin, with conventional *Umod^−/−^* knockout mice exhibiting increased vesicular accumulation of ROMK.^37^ Similarly, both global and tissue-specific ROMK knockout mice, as well as human Bartter-type patients with ROMK inactivating mutations, show defective urinary uromodulin secretion.^38^ It is, therefore, reasonable to assume that interaction between ROMK and uromodulin is required for their apical transport. Uromodulin has also been assigned a role in calcium and magnesium homeostasis. More specifically, urinary uptake of magnesium is fine-tuned by the DCT via an active transcellular pathway involving the magnesium channel TRPM6 (transient receptor potential melastatin 6).^39^ Studies with conventional *Umod^−/−^*knockout mice demonstrated lowering of TRPM6 in the DCT, while secreted uromodulin enhances the receptors’ cell surface abundance and current density.^40^ A similar effect was observed on transcellular calcium reuptake by the TRPV5/6 (transient receptor potential cation channel subfamily V member 5 and 6) located in the second part of the DCT as well as the connecting tubules.^41,42^ The complex interactions of uromodulin with ion channels and receptors of the TAL and DCT, together with their functional significance, have recently been reviewed.^43^

## Other Functions of Uromodulin

In addition to its involvement with renal ion handling, uromodulin has also been postulated to underly processes that protect against urinary tract infections by the nature of its 3-dimensional polymer meshwork which encapsulates and clears bacteria by urinary excretion.^25^ Evidence also suggests uromodulin inhibits the formation of kidney stones by inhibiting the aggregation of calcium oxalate and calcium phosphate.^44^ Moreover, in vitro studies with urinary uromodulin point towards an immunomodulatory role, whereby initially it was shown to inhibit T-cell and monocyte activity.^1,32^ However, it has also been demonstrated to activate various inflammatory cells such as neutrophils,^45^ macrophages,^46^ and dendritic cells.^47^ In contrast, circulating uromodulin appears to have anti-inflammatory properties, by attenuating renal and systemic oxidative stress by suppressing the activity of the nonselective calcium ion channel TRPM2 (transient receptor potential cation channel‚ subfamily M‚ member 2).^48^ Another study proposed inhibition of vascular calcification by serum uromodulin in chronic kidney disease (CKD).^49^ It is vital that the research into the functions of uromodulin continues to fully unlock its therapeutic potential.

## Role as a Marker of Kidney Health

Kidney health and cardiovascular health are tightly interlinked. Serum creatinine, cystatin C, and urea are conventional markers of renal excretory function, and their retention in the body can reflect a decrease in kidney function. These markers represent the glomerular filtration process and thus are late markers of renal injury; for example, about 50% of kidney function must be lost before a significant rise in serum creatinine can be detected.^50^ Other conventional markers of renal function include tubular injury markers, such as KIM1 (Kidney injury molecule-1) and NGAL (neutrophil gelatinase-associated lipocalin) and proteinuria and in particular albuminuria which typically indicates glomerular damage. In contrast, the expression and release of UMOD occurs only within the kidneys and therefore directly reflects the structural and functional condition of renal tubules and the interstitium.^51^

In the past 2 decades, uromodulin has been studied as potential predictive marker of renal transplant outcome, acute kidney injury, CKD, and cardiovascular disease. Higher levels of UMOD are associated with lower risk of developing end-stage renal disease in patients with CKD.^52–54^ Lower urinary UMOD levels were shown to be associated with rapid loss of estimated glomerular filtration rate (eGFR) and progression to end-stage renal disease in CKD patients within 1 year^52^ and in the long term (9–10 years).^53,54^ In patients undergoing cardiac surgery, preoperative lower urinary UMOD levels were found to be associated with higher postoperative risk of acute kidney injury both in adults^55,56^ and pediatric patients.^57^ More recently, it was shown in a small cohort of patients that levels of urinary UMOD with the external hydrophobic patch (polymerization-incompetent UMOD) were significantly lower in patients who develop acute kidney injury during hospitalization.^58^ A cluster of urinary peptides representing the polymerization-incompetent UMOD was shown to be increased in preeclamptic women.^59,60^

Over the past decade, the ease of measurement of circulating/serum UMOD has led to a large number of clinical association studies. There are a few important analytical differences between circulating and urinary UMOD. First, urinary UMOD polymerizes while circulating UMOD is a monomer; polymerization interferes with sample processing and storage, and urinary UMOD detection.^61^ Second, compared with urinary UMOD, circulating UMOD is more stable during storage at a wider range of temperatures and across freeze/thaw cycles.^27^ Third, urinary UMOD is ideally measured in 24 hours urine or, when measured in spot urine, it has to be normalized to urinary creatinine. In contrast, circulating UMOD can be measured as concentration and is strongly correlated with eGFR.^62^ It should be noted that normal values of circulating UMOD depend on sex and age.^27,63^

Changes in concentration of circulating UMOD can also be observed at different stages of CKD and add value to conventional biomarkers.^64–66^ For example, circulating UMOD concentrations are significantly higher in individuals without CKD (167.6 ng/mL) compared to individuals at all stages of CKD (stages 1–5: 111, 107, 71, 38, and 24.8 ng/mL, respectively).^64^ Circulating UMOD has been shown to be an effective parameter for monitoring kidney function in transplant patients. Higher pretransplant circulating UMOD levels were found to be associated with lower odds for delayed graft rejection,^67^ whereas in renal transplant recipients, lower circulating UMOD levels are associated with a higher risk of failure or rejection.^68,69^

In recent years, the clinical value of UMOD has extended beyond being a renal pathophysiology biomarker. In the elderly (Cooperative Health Research in the Region of Augsburg [KORA F4] F4 study; aged 62–81 years), lower circulating UMOD levels are associated with metabolic alterations, such as elevated triglycerides, reduced HDL (high-density lipoprotein) cholesterol, elevated blood pressure, and elevated fasting glucose.^70^ In the same age group, circulating UMOD was found to be inversely correlated with biomarkers of subclinical inflammation, such as C-reactive protein, IL (interleukin)-6, IL-22, IL-1 receptor antagonist, white blood cell count, tumor necrosis factor-α, superoxide dismutase-3, and myeloperoxidase.^70^

In addition, rare monogenic forms of human disease cause autosomal dominant tubulointerstitial kidney disease (ADTKD) characterized by a progressive decline in kidney function. UMOD-ADTKD disease is typically characterized by hyperuricemia, although with an absence of the hematuria or proteinuria normally associated with other renal disorders.^71^ The protein-coding mutations driving UMOD-ADTKD disease cause misfolding which allows the mutated uromodulin protein to accumulate in the ER, causing increased intracellular accumulation, ER-stress, and reduced uromodulin excretion.^72^ In an UMOD-ADTKD mouse model, it was shown that TAL cells of these animals had upregulation of Grp78 (78-kDa glucose-regulated protein), a marker or ER-stress, and additional upregulation of pathways indicating intracellular unfolded protein responses.^73^

## Role in Blood Pressure and Salt Sensitivity

### Association of Uromodulin With Blood Pressure

Family-based linkage studies and genome-wide association studies (GWAS) over the past 3 decades have given us the most detailed picture of the genetic architecture of blood pressure including 31 different genes harboring rare causal variants for monogenic blood pressure (BP) syndromes and over 1500 common single nucleotide polymorphisms (SNPs) associated with polygenic BP phenotypes.^74^ Among the plethora of common BP genetic variants, each with very small effects on blood pressure, is the *UMOD* promoter locus which has been the target of sustained multipronged epidemiological, functional genomic, and clinical studies. The discovery GWAS that first identified the promoter *UMOD* locus^75^ arrived on the back of GWAS of CKD that showed association of the same locus with eGFR and CKD,^76^ setting the scene for the challenge—is *UMOD* a BP or CKD gene? Linking the GWAS SNP (Figure 1) in the *UMOD* promoter region to uromodulin was relatively straightforward as it is exclusively expressed in the kidneys. Measurement of uromodulin in the urine provided early confirmation that the promoter SNPs (rs13333226, rs12917707, rs4494548 within an linkage disequilibrium block) were associated with urinary uromodulin.^75,77^ The minor allele of these SNPs are associated with a lower risk of hypertension,^75^ lower systolic and diastolic BP,^75,78,79^ increased estimated glomerular filtration rate,^80^ reduced urinary uromodulin,^75,77^ and lower fractional excretion of endogenous lithium.^75^ These associations are likely a mixture of direct causation, reverse causation, and confounding, which leads to the next post-GWAS challenge, establishing causality. A high-level review of these associations does not indicate a single common pathway mediating all these effects—for instance, the blood pressure association may be either a cause or consequence of renal perturbations.

Definitive evidence that uromodulin is independently associated with blood pressure comes from mendelian randomization studies, which are powerful statistical methods that infer causality from population genetic studies. Here, utilizing urinary uromodulin GWAS SNPs as exposures and GWAS SNPs of blood pressure and renal function as outcomes, Ponte et al^81^ showed that each 1-mg genetically predicted urinary uromodulin/g creatinine was associated with 1 mL/min/1.73m^2^) lower eGFR, 6% higher odds of having CKD,^81^ 0.11 mm Hg higher systolic BP, and 0.09 mm Hg higher diastolic BP.^81^ Quantifying the independent effect of uromodulin on BP and eGFR was carried out using bidirectional and multivariable mendelian randomization to show that 28% of uromodulin’s total effect on BP was mediated by eGFR, with the remainder due to the direct effect.^81^

There are, however, contrasting results observed regarding urinary UMOD and blood pressure. A positive correlation between urinary UMOD and mean arterial pressure was observed in elderly hypertensive patients,^82^ whereas in the Health, Aging & Body Composition study, participants with higher levels of urinary UMOD had lower systolic blood pressure.^53^ In contrast to urinary UMOD, a consistent inverse relationship between circulating UMOD and hypertension was observed in various studies.^82–85^

### Association of Uromodulin With Sodium Homeostasis

An independent role of uromodulin on sodium homeostasis thereby pointing to a causal role for uromodulin on BP regulation came from two complementary experiments in *Umod*^−/−86^ and over-expressing^87^ mice models as mentioned below. These studies along with the demonstration of a shift-to-left of the pressure-natriuresis curve^86^ in *Umod*^−/−^ mice offer compelling evidence that uromodulin had a causal role in BP regulation through perturbations of sodium homeostasis and tubular function independent of its effect on glomerular filtration. Further support for uromodulin influencing sodium homeostasis through tubular mechanisms comes from general population studies where higher urinary uromodulin concentrations have been shown to associate with higher urinary sodium, chloride, and potassium excretion and osmolality.^88^

Few interventional studies investigated the relationship between blood pressure, uromodulin, and salt. The Dietary Approaches to Stop Hypertension (DASH)-sodium trial showed that higher urinary UMOD levels were not associated with an increase in blood pressure.^89^ Whereas a study on family-based Chinese cohort identified *UMOD* SNPs associated with the blood pressure responses to salt interventions.^90^ They also show that high salt intake reduced the 24-hour urinary UMOD excretion.^90^

### Mechanistic Approaches to Study Blood Pressure and Salt Sensitivity (Preclinical Models)

**Figure 2.**
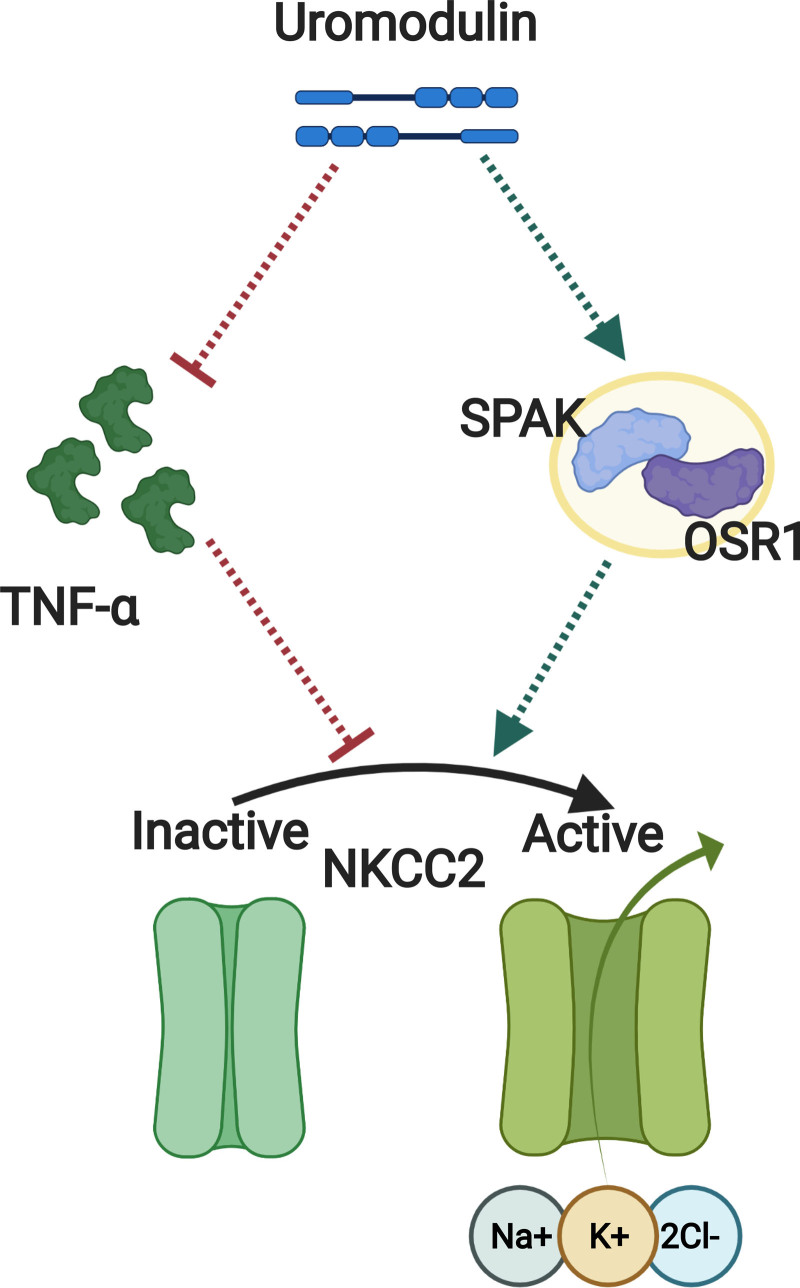
**Uromodulin regulates salt reabsorption via NKCC2 (Na^+^-K^+^-2Cl^−^ cotransporter).** Two mechanistic approaches studied through preclinical models show that uromodulin regulates NKCC2 (Na^+^-K^+^-2Cl^−^ cotransporter) via activation (phosphorylation) of SPAK (STE20/SPS1–related proline/alanine-rich kinase) and OSR1 (oxidative stress response 1 kinase), and inhibition of TNF (tumor necrosis factor)-alpha. The red and green dotted arrow represents indirect signaling pathway for inhibition and activation, respectively.

Preclinical models contribute to the understanding of the cause and progression of human disease mechanisms which is crucial for clinical translation. It seems clear that UMOD associates with BP through several pathways contributing to varying effect sizes. Two distinct but early complementary papers have been published providing causative mechanisms, with both articles differing in their approach and interpretation suggesting that UMOD affects NKCC2 protein quantity or activity, although the drivers of this relationship have not yet been fully elucidated and are likely multifaceted (Figure 2).^86,87^ Trudu et al^87^ generated a human overexpressing *UMOD* transgenic mouse (Tg*Umod*^wt/wt^) on an FVB/N (Friend Virus B NIH Jackson) background, which displayed increased phosphorylation of NKCC2 at two known activation sites (Threonine 96 and Threonine 101) and salt-sensitive hypertension. These transgenic over-expressers were shown to have increased baseline blood pressure and were more sensitive to the removal of dietary NaCl than their wild-type counterparts. In stable transfectants of NKCC2 in renal cells, activation of the cotransporter was observed in wild-type uromodulin cotransfectants but not in solubilized uromodulin cotransfectants, implying that the action of uromodulin in relation to NKCC2 activation is dictated by cellular membrane–anchored uromodulin.^87^ Further investigation highlighted the modulation of NKCC2 activation by uromodulin in their Tg*Umod*^wt/wt^ mouse model was mediated in part by increased activation of SPAK (sterile 20 [Ste20/SPS1] related proline/alanine rich kinase) and OSR1 (oxidative stress response 1 kinase)—themselves known regulators of NKCC2 phosphorylation. Thus, Trudu et al^87^ provided a clear causative mechanistic relationship between UMOD and blood pressure regulation, through uromodulin associating with increased phosphorylation/activity of NKCC2 via SPAK and OSR1. Additionally, it was shown that loop-diuretic treatment was significantly more effective at reducing blood pressure in Tg*Umod*^wt/wt^ mice versus wild-type counterparts.^87^

**Figure 3.**
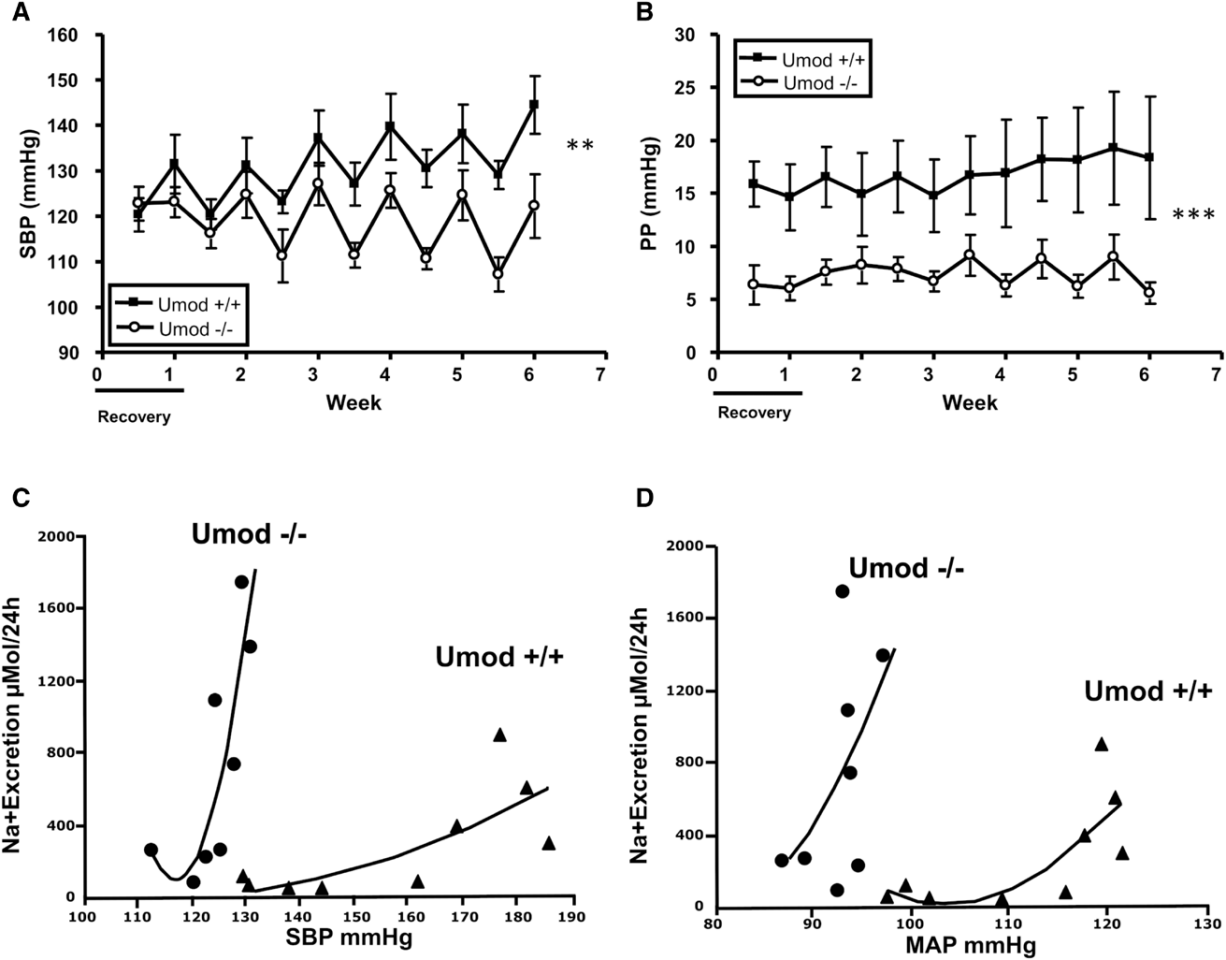
***Umod*^−/−^ knockout mice are not sensitive to NaCl-induced changes in blood pressure.** Wild-type (WT) mice have significantly increased (**A**) systolic blood pressure (SBP) and (**B**) pulse pressure (PP) compared with the *Umod*^−/−^. **C** and **D**, There is a leftward shift in the chronic renal function curves (SBP, mean arterial pressure [MAP]) in *Umod*^−/−^ mice compared with the WT animals. Adapted from Graham et al^86^ with permission. Copyright ©2014, Wolters Kluwer Health, Inc.

Graham et al^86^ employed a knockout-driven approach, using homozygous uromodulin knockout mice on an Sv129 background (*Umod*^−/−^), which significantly lowered systolic blood pressure on a normal chow diet and showed resistance to additional salt (NaCl) added to drinking water compared to wild-type mice with similar salt intake. These studies along with the demonstration of a shift-to-left of the pressure-natriuresis curves in *Umod*^−/−^ mice offer compelling evidence that uromodulin has a causal role in BP regulation (Figure 3) through perturbations of sodium homeostasis and tubular function independent of its effect on glomerular filtration.^35,86^ Reduced levels of NKCC2 mRNA expression in *Umod*^−/−^ knockout animals were also identified and urinary TNF (tumor necrosis factor)-α excretion was significantly increased in the presence of dietary salt suggesting TNF-α is central to this regulation. Consistent with this finding, TNF-α has been shown to affect the bioavailability and function of NKCC2.^91^ In TAL cells of the TNF-α^−/−^ mice both protein and mRNA expression of NKCC2 were significantly upregulated suggesting TNF-α acts as an endogenous inhibitor of NKCC2. In addition, NKCC2 activity has been shown to respond to NaCl levels, which can be attenuated by TNF-α, with low salt diet leading to increased phosphorylation of NKCC2 and upregulation of NKCC2. This upregulation was subsequently diminished by treatment with endogenous TNF-α.^92^ Although age of the knockout animals is likely to have an effect on blood pressure findings.^93,94^ Additional mouse models have been generated that will facilitate research into the TAL segment of the nephron using the conserved uromodulin promoter segment across various species.^11^

Other mouse models that subsequently cause an accumulation of intracellular UMOD include the Hepsin (*Hpn*^−/−^) knockout mouse indicating that appropriate processing of UMOD is critical for its function.^95^ It was shown that hepsin-mediated processing of UMOD is important for salt sensitivity. These *Hpn*^−/−^ knockout mice accumulate UMOD in ER and apical membrane of TAL cells and 2% salt loading for 2 months further increased the accumulation of UMOD in the kidney leading to ER-stress and distal tubular damage.^95^

Our recent study aimed to dissect the role of salt and blood pressure on UMOD excretion in a rat model. Interestingly, we found that high salt loading decreased UMOD excretion both in normotensive (Wistar Kyoto rats) and chronic hypertensive (Stroke Prone Spontaneously Hypertensive rats) with and without antihypertensive treatment. Further salt loading increases intracellular UMOD accumulation in chronic model of hypertension with increases in kidney injury markers (KIM-1 and NGAL).^96^

### Linking Blood Pressure and Salt Sensitivity: Future Trials

**Figure 4.**
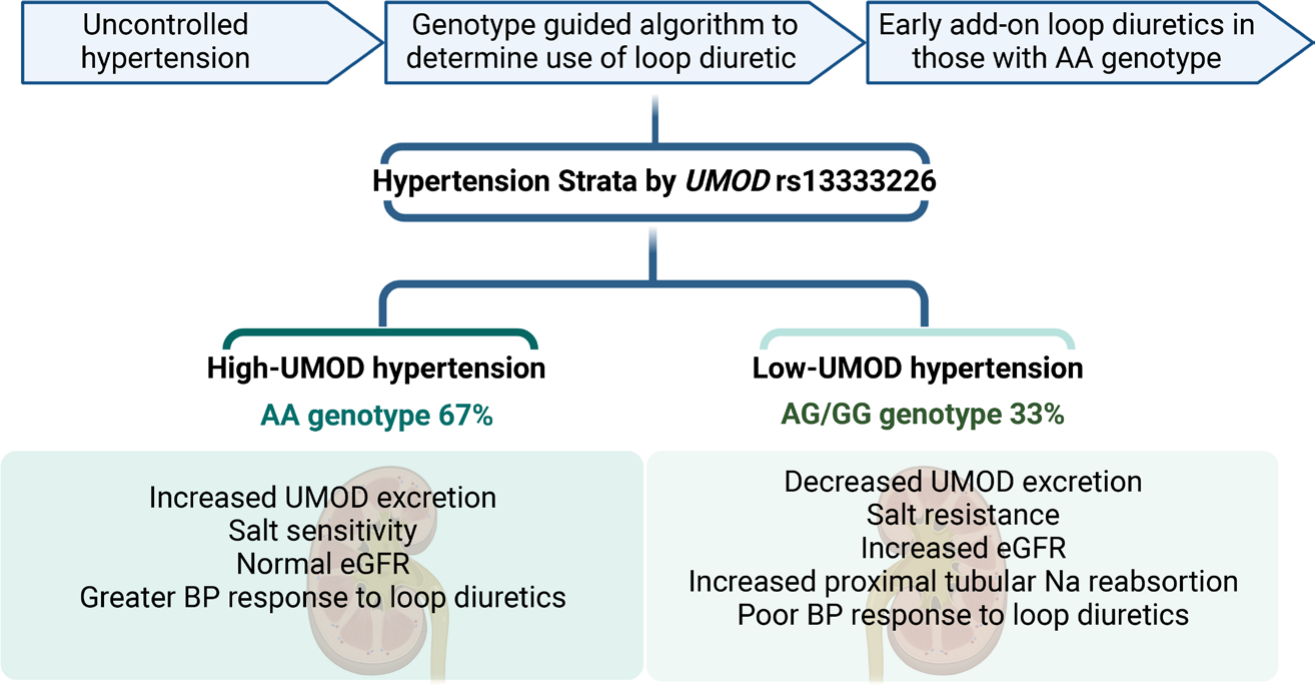
**Uromodulin in precision medicine.** The figure represents the rationale for the clinical trial (https://www.clinicaltrials.gov; Unique identifier: NCT03354897) for understanding the influence of *UMOD* variant on blood pressure (BP) for personalized treatment. eGFR indicates estimated glomerular filtration rate.

As discussed above, this direct effect of uromodulin on BP is likely mediated through renal sodium handling evoking a putative interaction with NKCC2. Loop-diuretics are inhibitors of NKCC2 offering an opportunity to establish a link between uromodulin, NKCC2, and blood pressure through interventional studies.^97^ Treatment with furosemide, a loop-diuretic, significantly enhanced natriuresis and reduced BP levels both in the transgenic mice overexpressing UMOD and in treatment-naïve hypertensive individuals homozygous for the uromodulin increasing allele.^87^ Definitive evidence in human subjects of UMOD-NKCC2 interaction and consequent effect on salt sensitivity is currently being tested through a prospective genotype-directed clinical trial (https://www.clinicaltrials.gov; Unique identifier: NCT03354897).^97^ This trial is the first precision medicine trial for hypertension, which is designed to test the hypothesis that hypertensive patients will show differential BP response to loop-diuretics based on their uromodulin genotype. The primary objective is to test whether hypertensive subjects with uncontrolled BP possessing the uromodulin increasing rs13333226 AA genotype will be better responders to loop-diuretics (torasemide) than those possessing either one or two copies of the G allele (Figure 4).^97^ Recruitment for this trial is completed and results are expected in the second quarter of 2022. If the results of this trial confirm the primary hypothesis, then the link between UMOD and NKCC2 influencing renal sodium handling is further strengthened. From a translational perspective, this would support prioritizing loop-diuretics for hypertensive patients who are homozygous for the *UMOD* BP-increasing genotype.

## Summary

Our knowledge of the role of uromodulin in the regulation of blood pressure and kidney function has vastly increased over the last decade. However, there are still numerous unanswered questions. The causal relationship between urinary uromodulin and blood pressure and association with kidney function has opened the possibility for uromodulin as an excellent biomarker for precision medicine interventions. It is important to note that the sex differences in UMOD physiology and pathophysiology have not been studied adequately thus far, and would require more attention in the future. Further research into molecular insights on regulation, secretion, and post-translational modifications will make uromodulin a potential target for studies to discover new druggable targets for hypertension and CKD. This is exemplified by the genotype-directed clinical trial (Figure 4),^97^ which might transform and widen our treatment modalities for difficult-to-treat hypertension due to in-depth understanding of the uromodulin function dependent on genetic stratification.

## Article Information

### Acknowledgments

We acknowledge the contributions of our previous and present staff and students’ work on the uromodulin project, especially, Lesley Graham, Simon Fisher, and Manshi Zhou. Figures 1, 2, and 4 were created on Biorender.com. Figure 3 is recreated with permission from our previous publication Graham et al.^86^

### Sources of Funding

P. Boder is supported by a British Heart Foundation PhD studentship (FS/18/58/34179). M.W. McBride is supported by FS/17/63/33485. S. Padmanabhan, C. Delles, and A.F. Dominiczak are supported by a British Heart Foundation Centre of Research Excellence Award (RE/18/6/34217). S. Padmanabhan is funded by the Medical Research Council (MR/M016560/1; AIM-HY Study), the British Heart Foundation (BHF CS/16/1/31878), and Heart Research UK (RG2690/21/24). A.F. Dominiczak and S. Padmanabhan acknowledge funding from the UKRI Strength in Places Fund (SIPF00007/1).

### Disclosures

None.
